# Discordance between MTB/RIF and Real-Time Tuberculosis-Specific Polymerase Chain Reaction Assay in Bronchial Washing Specimen and Its Clinical Implications

**DOI:** 10.1371/journal.pone.0164923

**Published:** 2016-10-19

**Authors:** Yong Suk Jo, Ju-Hee Park, Jung Kyu Lee, Eun Young Heo, Hee Soon Chung, Deog Kyeom Kim

**Affiliations:** 1 Division of Pulmonary and Critical Care Medicine, Department of Internal Medicine, Seoul National University College of Medicine, Seoul, Republic of Korea; 2 Division of Pulmonary and Critical Care Medicine, Department of Internal Medicine, Seoul Metropolitan Government-Seoul National University Boramae Medical Center, Seoul National University College of Medicine, Seoul, Republic of Korea; Universidad Nacional de la Plata, ARGENTINA

## Abstract

The prevalence and clinical implications of discordance between Xpert MTB/RIF assays and the AdvanSure TB/NTM real-time polymerase chain reaction (PCR) for bronchial washing specimens have not been studied in pulmonary TB (PTB) patients. The discordant proportion and its clinical impact were evaluated in 320 patients from the bronchoscopy registry whose bronchial washing specimens were tested simultaneously with Xpert MTB/RIF and the TB/NTM PCR assay for three years, and the accuracy of the assays, including the sensitivity, specificity, positive predictive value (PPV), and negative predictive value (NPV), were studied. The clinical risk factors for discordance and false positivity of assays were also studied. Among 130 patients who were clinically diagnosed with PTB, 64 patients showed positive acid-fast bacilli culture results, 56 patients showed positive results in molecular methods and clinician diagnosed PTB without results of microbiology in 10 patients. The sensitivity, specificity, PPV, and NPV were 80.0%, 98.95%, 98.1%, and 87.9%, respectively, for Xpert MTB/RIF and 81.5%, 92.6%, 88.3%, and 88.0%, respectively, for TB/NTM PCR. The discordant proportion was 16.9% and was higher in culture-negative PTB compared to culture-confirmed PTB (24.3% vs. 9.4%, p = 0.024). However, there were no significant differences in the clinical characteristics, regardless of the discordance. The diagnostic yield increased with an additional assay (7.7% for Xpert MTB/RIF and 9.2% for TB/NTM PCR). False positivity was less common in patients tested with Xpert MTB/RIF (1.05% vs. 7.37%, p = 0.0035). No host-related risk factor for false positivity was identified. The Xpert MTB/RIF and TB/NTM PCR assay in bronchial washing specimens can improve the diagnostic yields for PTB, although there were considerable discordant results without any patient-related risk factors.

## Introduction

Tuberculosis (TB) is one of the most critical infectious diseases worldwide. [1,2] In TB-prevalent areas, early and accurate diagnosis is crucial for the timely initiation of treatment and prevention of lung deterioration and disease transmission. An acid-fast bacilli (AFB) smear and culture is the gold standard for TB diagnosis. An AFB smear has high specificity and short turnaround time, but the low sensitivity is a challenge. Although an AFB culture has high sensitivity and specificity, it takes more than 4 weeks. [3–5] Therefore, several molecular diagnostic methods for early detection of *Mycobacterium tuberculosis* have been developed.

Real-time polymerase chain reaction (PCR) assay for M. TB including commercial assay kits has high specificity and sensitivity as well as a short turn-around time. [6–8] It can also minimize contamination by allowing direct use of the clinical specimen in a closed system. [9] Another molecular biological method Xpert MTB/RIF assay is also a highly sensitive and specific diagnostic tool for TB diagnosis, and furthermore, it can detect rifampin resistance simultaneously within 2 hours. [10] Despite highly sensitive and specific molecular methods being widely used in clinical practice to detect TB, there are few studies that show which assay is more accurate or whether they are complementary. [11]

Although sputum is a simple specimen used for diagnosis of pulmonary TB (PTB), there is a risk of contamination during sample collection. Additionally, if the PTB is not severe, culture positivity is normally reported for weeks afterward despite smear negativity. [12,13] In cases with minimal lesions or during the early stage of PTB, bronchoscopic examination could improve the diagnostic accuracy and aid in rapid diagnosis following advance treatment of PTB. [14,15] Therefore, respiratory specimens obtained from bronchoscopy, such as washings or lavage, are tested using TB PCR; nevertheless, the diagnostic accuracy and discordance of various TB PCR assays for bronchial washing specimens are rarely compared.

Additionally, even though these two TB PCR assays are highly sensitive and specific, there is an issue of false positives [16–18]. Especially in TB endemic areas, use of PCR for TB diagnosis is frequent, because PTB can mimic community-acquired pneumonia and may increase the possibility of false positives. This may pose a serious problem for patients who should take anti-TB drugs for six months based on the results of PCR. Therefore, it is necessary to compare the diagnostic accuracy and concurrence between Xpert MTB/RIF and real-time PCR assay as well as evaluate their clinical impact in TB-prevalent areas. It is also critical to ascertain the clinical risk factors that may lead to discordance in these two assays.

This study aimed to evaluate the diagnostic discordance between Xpert MTB/RIF and real-time TB PCR assay using a bronchial washing specimen and determine the clinical implication of the discordance in the two tests.

## Methods

### Study design and respiratory specimen

We reviewed the medical records of patients who were enrolled in a bronchoscopy registry of Boramae Medical Center, a referral municipal and Seoul National University affiliated hospital in South Korea, who simultaneously underwent Xpert MTB/RIF and real-time TB PCR assay using bronchial washing specimens from May 1, 2013 to February 28, 2016.

We used a flexible fiberoptic bronchoscope with a 5.9-mm diameter (model BF-200 or BF-1T240, Olympus Optical Co, Tokyo, Japan), and the procedures were performed by full-time faculty staff in the Pulmonology department. After inspecting all visible bronchial trees, samples were collected from the lung segment or subsegment that showed abnormal lesions suggestive of active PTB on chest computed tomography (CT). Whether bronchial washing or BAL was performed depended on the clinician’s decision to increase the diagnostic yield.

Diagnosis of PTB was defined not only by culturing *M*. *tuberculosis* from bronchoscopically obtained specimens (*i*.*e*., culture-confirmed TB in this analysis) but also by clinically diagnosed PTB for patients treated with anti-TB drugs included for analysis (*i*.*e*., culture-negative TB). We defined PTB by primarily on the results of AFB smear and culture but either one of nucleic acid amplification (NAA) test positivity with highly suspected to having PTB on the basis of radiological and respiratory symptoms also regarded as PTB patients. On the contrary, even patients who has positive results on either one of the NAA test but clinician do not highly suspect PTB and keep going observation at outpatient clinic without anti-TB drugs and showed no clinical deterioration but improvement defined as false positivity on NAA tests.

The laboratory facilities were qualified by a clinical laboratory accreditation program in Korea. This study was approved by the Institutional Review Board (IRB) of Boramae Medical Center (IRB no. 26-2016-45) and waived the need for informed consent because no patient at risk. Medical records were anonymized and de-identified prior to access by the researchers.

### Real-time TB/NTM PCR

The AdvanSure TB/NTM real-time PCR (AdvanSure PCR; LG Lifescience, Korea) is a real-time PCR kit that targets IS6110 specific for *M*. *tuberculosis* complex and *rpoB* gene specific for AFB, and thus, it can detect TB and non-tuberculous mycobacterium (NTM) as well. We followed manufacturer’s instruction of processing samples and PCR reaction. After mixing 5-6mL of bronchial washing fluid with an equal amount of 1N NaOH, vortexing for 30 seconds and centrifugation for 20 minutes at 3000 rpm were performed. The pellet was mixed with 1mL of a buffer and vortexed again. Then, the mixture was centrifuged for 5 minutes at 7000 rpm. The sequential processes were repeated three times. After removing the supernatant, the sample was boiled for 10 minutes and centrifuged for 3 minutes at 13,000 rpm. Finally, about 1–2 μL of final supernatant was taken for the PCR reaction with PCR mixture and primer sequences (IS6100 and *rpoB* gene) with probe mixed. Real time PCR was performed by using the AdvanSure TB/NTM real-time PCR Kit and SLAN real-time PCR detection system (AdvanSure PCR; LG Lifescience, Korea) was used to measure fluorescence formed during PCR process. Cycle thresholds (C_T_) lower than 35 means positive after observing signal of wave length from three channels (*M*. *tuberculosis* complex, mycobacteria, internal control) As manufacturer’s instruction, if the value of C_T_ of *rpoB* is higher than the value of C_T_ of IS6110 as judged as TB, and if the value of C_T_ of *rpoB* lower than 35 is judged as NTM.

### Xpert MTB/RIF assay

The Xpert MTB/RIF (Cepheid, Sunnyvale, CA, USA), rapid, automated cartridge-based nucleic acid amplification test using RT-PCR for the TB-specific *rpoB* gene can detect TB and rifampin resistance simultaneously, and the result is available in less than 2 hours. The mixture of sample with 2 ml Xpert sample reagent incubated for 15 min at room temperature, then transferred into an Xpert cartridge and inserted into the GeneXpert instrument. C_T_ of 5 rpoB gene probes automatically reported the presence of *M*.*tuberculosis*.

These two molecular methods were performed and interpreted according to the manufacturer’s instructions.

### AFB smear and culture

AFB smears were performed using auramine-rhodamine fluorescent staining and confirmed by Ziehl-Neelsen staining. The sediment was cultivated on 3% Ogawa solid media for 8 weeks in 5–10% CO_2_ incubators as well as in BACTEC^™^ MGIT^™^ system, a liquid culture system, for 6 weeks. Once cultured, isolation of MTB was confirmed using the Gen-Probe^®^ method (Gen-Probe, San Diego, CA, USA). [19]

### Statistical analysis

Descriptive data are expressed as mean ± SD or number (%), unless otherwise specified. Chi-square test and Fisher’s exact tests were used to compare the categorical variables, and Student’s t-test was employed for comparison of continuous variables. The sensitivity, specificity, positive predictive value (PPV), and negative predictive value (NPV) were calculated using the 95% confidence intervals (CI) to determine the diagnostic accuracy of TB/NTM PCR and Xpert MTB/RIF assay. In 10 patients, bronchoscopy was performed repeatedly depending on their clinical conditions. In this analysis, each examination was regarded as an independent case.

All analyses were two-sided and performed at a 0.05 significance level, unless otherwise noted. A P-value < .05 was considered statistically significant. All analyses were carried out using STATA version 13.1 (StataCorp, College Station, Texas, US).

## Results

### Clinical characteristics of study population

**Fig 1:**
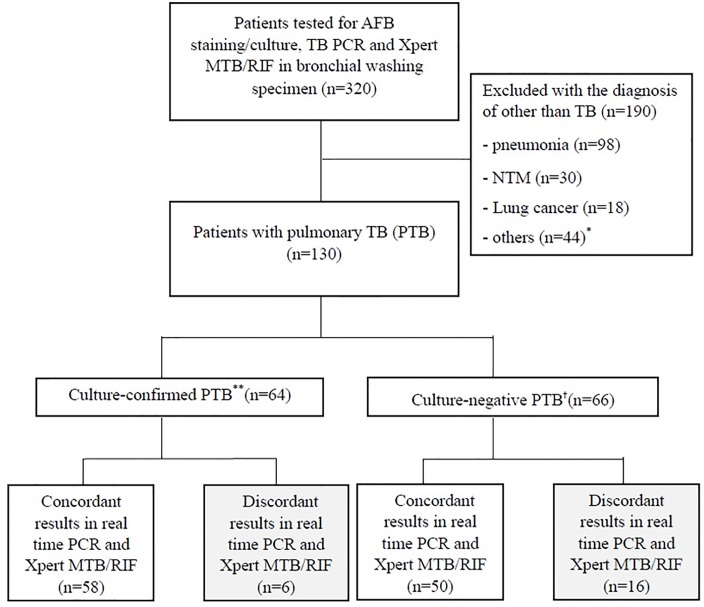
Study flow. Abbreviation: AFB = acid-fast bacilli, TB = tuberculosis, PCR = polymerase chain reaction, NTM = non-tuberculosis mycobacterium. * other diseases (number): bronchiectasis(3), empyema(3), benign bronchial stenosis(2), anthracofibrosis(1), fungal ball(1), lung abscess(1), diffuse panbronchiolitis(1). ** Pulmonary TB (PTB) with growth of *M*. *tuberculosis* in culture study. ^†^ PTB without growth of *M*. *tuberculosis* which was diagnosed with PCR for TB or clinical decision.

Bronchoscopic specimens from 320 patients were subjected to AFB staining and culture, Xpert MTB/RIF, and TB/NTM PCR assay simultaneously. Among them, 130 patients were diagnosed with PTB. Sixty-four patients had culture-confirmed PTB diagnosed microbiologically with the growth of *M*. *tuberculosis* and 66 patients had culture-negative PTB diagnosed with either Xpert MTB/RIF or TB/NTM PCR assay and/or a clinical decision based on the clinical course, therapeutic response, and radiologic findings. (Fig 1)

Demographic characteristics showed no difference in both the groups, except that patients with a diagnosis other than TB had lower BMI. Radiographic findings revealed that cavity and clustered nodules were more common in PTB. The extent of the lung involved was more extensive in PTB patients. (Table 1) Among patients with PTB, 10 (7.69%) had mutation on *rpoB* gene on Xpert MTB/RIF assay and were treated with second-line anti-TB drugs. These patients finally showed multi-drug resistant TB in the final drug sensitivity test.

**Table 1:** Clinical characteristics of patients tested for AFB stain/culture, TB PCR and Xpert MTB/RIF assay in bronchial washing specimen^a^.

Characteristics, N (%)	Patients with pulmonary TB (n = 130)	Patients with the diagnosis of other than TB^b^ (n = 190)	P value
Sex, male	84 (64.62)	126 (66.32)	0.75
Age, years	61.99±16.90	64.64±17.26	0.80
Body mass index (BMI), kg/m^2^,	22.17±13.81	20.74±4.51	0.00
Previous history of pulmonary TB	41 (31.54)	47 (24.74)	0.18
Smoking status			0.29
Never smoker	54 (41.54)	89 (47.09)	
Ex-smoker	81 (24.81)	52 (27.51)	
Current smoker	68 (21.32)	35 (18.52)	
Smoking amount, pack-year	15.42±20.00	16.27±21.74	0.34
Comorbidities			
Diabetes mellitus	23 (17.69)	29 (15.26)	0.56
Chronic kidney disease	1 (0.77)	5 (2.63)	0.23
HIV/AIDS	0 (0)	1 (0.53)	0.41
Malignancy	11 (20.37)	41 (15.41)	0.37
Gastrectomy	7 (5.38)	7 (3.68)	0.47
Radiologic findings			
Cavity	30 (23.26)	21 (11.05)	0.01
Consolidation	75 (57.69)	96 (50.53)	0.34
Clustered nodules	86 (66.15)	94 (49.47)	0.01
Ground glass opacity	19 (14.62)	38 (20.00)	0.22
Pleural effusion	17 (13.08)	14 (7.37)	0.17
Atelectasis	18 (13.85)	24 (12.63)	0.68
Extent of involved lung^c^, number of region	2.30±1.73	2.10±1.44	0.02

^a^Data are presented in number (%) or mean ± standard deviation.

^b^ Pneumonia (98, 51.6%), NTM(30, 15.8%), lung cancer (18, 9.5%), bronchitis and bronchiolitis(17, 8.9%), others (27, 14.2%)

^c^ Number of involved regions when the lung was divided in six regions.

### Diagnostic accuracy of Xpert MTB/RIF and real-time TB/NTM PCR assay

The sensitivity of the TB/NTM PCR for diagnosis of PTB based on culture positivity was 85.94% (95% CI, 74.98–93.364) and the specificity was 74.61% (95% CI, 68.82–79.82), while PPV was 45.83% (95% CI, 36.71–55.17) and NPV was 95.50 (95% CI, 91.63–97.92).

In comparison, the sensitivity of the Xpert MTB/RIF assay was 92.19% (95% CI, 82.70–97.41) and specificity was 81.64% (95% CI, 76.34–86.19), while PPV was 55.66% (95% CI, 45.69–65.31) and NPV was 97.66% (95% CI, 94.63–99.24). We also analyzed those parameters based on all cases of clinically diagnosed PTB regardless of AFB culture positivity. (Table 2)

**Table 2:** Diagnostic accuracy of TB PCR and Xpert MTB/RIF assay for the diagnosis of pulmonary TB among AFB culture negative or positive patients.

	TB PCR		Xpert MTB/RIF		TB PCR^a^		Xpert MTB/RIF^a^	
	n/N	% (95% CI)	n/N	% (95% CI)	n/N	% (95% CI)	n/N	% (95% CI)
Sensitivity	55/64	85.94 (74.98–93.36)	59/64	92.19 (82.70–97.41)	106/130	81.54 (73.79–87.80)	104/130	80.00 (72.08–86.50)
Specificity	65/256	74.61 (68.82–79.82)	209/256	81.64 (76.34–86.19)	176/190	92.63 (87.95–95.91)	188/190	98.95 (96.25–99.87)
PPV	55/120	45.83 (36.71–55.17)	59/106	55.66 (45.69–65.31)	106/120	88.33 (81.20–93.47)	104/106	98.11 (93.35–99.77)
NPV	191/200	95.50 (91.63–97.92)	209/214	97.66 (94.63–99.24)	176/200	88.00 (82.67–92.16)	188/214	87.85 (82.71–91.91)

^a^ Analysis included cases diagnosed PTB based on clinical and radiological means regardless of AFB culture positivity

AFB, acid-fast bacilli; NPV, negative predictive value; PCR, polymerase chain reaction; PPV, positive predictive value; TB, tuberculosis; CI, confidence interval.

### Discordance proportion and comparison of the clinical characteristics between Xpert MTB/RIF and real-time TB/NTM PCR assay

Among the 130 patients with PTB, 22 (16.9%) showed disagreement on diagnosis of TB based on Xpert MTB/RIF (9.4%) and TB/NTM PCR assay (24.2%) (Table 3). The discordance was significantly higher with culture-negative PTB (p = 0.024).

**Table 3:** Discordance between real time TB PCR and Xpert MTB/RIF assay in bronchial washing specimen collected from patients treated with pulmonary TB*.

Type of assay		Real time TB PCR			
		Culture-confirmed PTB		Culture-negative PTB	
		positive	negative	positive	negative
Xpert MTB/RIF assay	Positive	54 (84.4)	5 (7.8)	40 (60.6)	5 (7.6)
	Negative	1 (1.6)	4 (6.2)	11 (16.7)	10 (15.1)

*Data are presented in number (%).

Among patients with culture-confirmed PTB, 6 showed discordant results in both tests, while discordant results were more common in patients with culture-negative PTB (16 patients). In culture-confirmed PTB, the number of patients who were Xpert MTB/RIF-positive and TB/NTM PCR negative (5 patients, 7.8%) was higher than patients who were Xpert MTB/RIF negative and TB/NTM PCR positive (1 patient, 1.6%) (Table 3), whereas in culture-negative PTB, the number of patients who were Xpert MTB/RIF-negative and TB/NTM PCR—positive (11 patients, 16.7%) was higher than patients who were Xpert MTB/RIF-positive and TB PCR negative (5 patients, 7.6%).

When we reviewed the demographic and radiographic variables as well as microbiological load which represented by positive result of AFB smear and comorbid diseases between the concordant and discordant groups, there was no significant risk factor that indicated discordance of the two assays in this population.

Even in multivariate analysis, adjusting for age, sex, smoking status, history of PTB, DM, and radiographic findings, including cavity on chest X-ray and extent of lung lesion, we were not able to find any risk factor associated with the discordance between the two molecular biological methods.

### Diagnostic yield and discordance proportion according to the order of using real-time TB/NTM PCR and Xpert MTB/RIF assay

Even though two assays were performed simultaneously during the study period, we calculated the additional diagnostic yield when each diagnostic test was applied in order.

**Fig 2:**
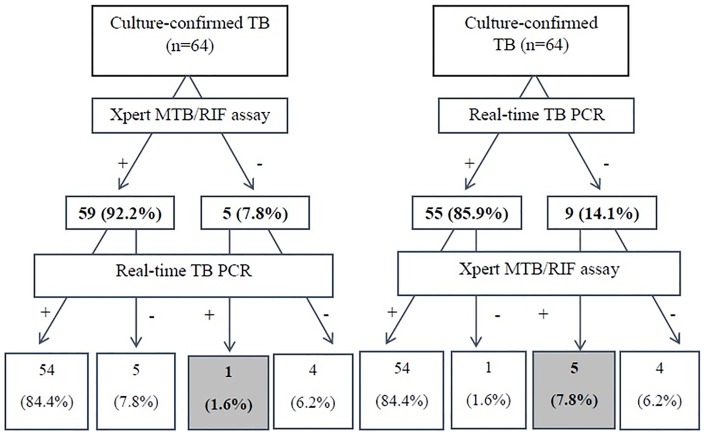
Diagnostic probability of TB according to the application order of real time TB/NTM PCR or Xpert MTB/RIF assay in patients with culture confirmed PTB.

**Fig 3:**
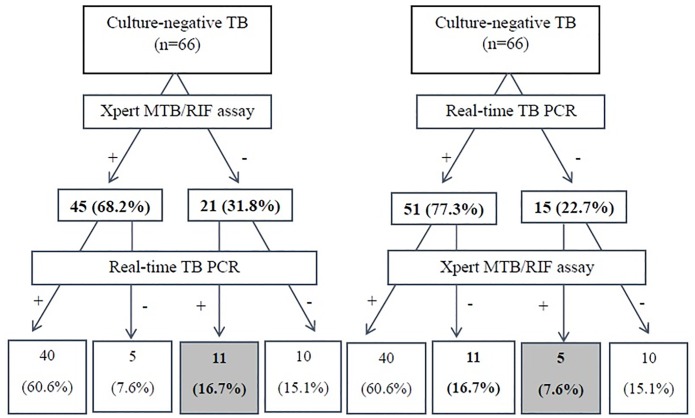
Diagnostic probability of TB according to the application order of real time TB/NTM PCR or Xpert MTB/RIF assay in patients with culture-negative PTB.

When the Xpert MTB/RIF assay was performed first, the number of PTB patients identified were higher than those with TB/NTM PCR (92.2% vs. 85.9%) and the subsequently performed TB/NTM PCR and Xpert MTB/RIF assay picked out 1 and 5 more PTB patients (1.6% vs. 7.8%), in culture-confirmed PTB patients. On the contrary, performing TB/NTM PCR first allowed detection of a higher number of PTB patients than the Xpert MTB/RIF assay (77.3% vs. 68.2%) and then each Xpert MTB/RIF assay and TB/NTM PCR identified 5 and 11 additional PTB patients (7.6% vs. 16.7%) in culture-negative PTB patients. (Figs 2 and 3)

The difference in additional yields of applying the other assay in patients with a negative result with the initial test was not statistically different (p > 0.05). The diagnostic yield and agreement of the two tests in all patients with PTB was shown in S1 Fig.

### Clinical characteristics of patients with false-positive results in real-time TB/NTM PCR or Xpert MTB/RIF

Among 190 patients diagnosed with PTB, 16 (8.4%) showed positivity on either TB/NTM PCR or Xpert MTB/RIF assay, i.e., false positivity. A majority of them (14 patients, 87.5%) showed false positivity in real-time TB/NTM PCR assay while 2 patients (12.5%) showed then same in Xpert MTB/RIF assay (Table 4). When we reviewed their clinical characteristics, 5 patients (31.3%) with a history of PTB did not show a cavity lesion on chest X-ray. Pneumonia was the most common final diagnosis, showing clinical and radiographic improvement without anti-TB treatment.

**Table 4:** Clinical characteristics of patients with false positivity in either real time TB PCR or Xpert MTB/RIF assay.

Patients No.	Age	Sex	Real-time TB/NTM PCR	Xpert MTB/RIF assay	Smoking status	History of previous TB	DM	Cavity on chest X-ray	Final diagnosis
1^a^	46	F	-	+	Never	-	-	-	Endobronchial metastasis from breast cancer
2^b^	95	F	-	+	Never	-	-	-	pneumonia
3	78	F	+	-	Never	-	Present	-	Bronchiolitis and bronchitis
4	76	M	+	-	Never	-	Present	-	Pneumonia
5 ^a^	74	M	+	-	Current	-	-	-	Lung cancer
6 ^b^	70	M	+	-	Ex-smoker	-	-	-	pneumonia
7 ^b^	68	M	+	-	Ex-smoker	Present	-	-	pneumonia
8 ^b^	68	M	+	-	Ex-smoker	Present	-	-	pneumonia
9 ^b^	71	M	+	-	Never	-	-	-	pneumonia
10 ^b^	95	F	+	-	Never	-	-	-	pneumonia
11	25	F	+	-	Never	Present	-	-	Inflammatory nodule
12 ^b^	52	M	+	-	Current	-	-	-	Pneumonia
13	82	M	+	-	Ex-smoker	-	-	-	NTM
14	43	M	+	-	Unknown	Present	-	-	Previous TB sequelae
15^c^	63	M	+	-	Ex-smoker	Present	-	-	Lung cancer
16 ^c^	79	M	+	-	Ex-smoker	-	-	-	Lung cancer

^a^ Repeated bronchoscopy revealed negative on TB/NTM PCR

^b^ Patients were treated with antibiotics under clinically diagnosed with pneumonia, and showed improvement as a result.

^c^ Clinicians regard positive result on TB/NTM PCR as a false positive under the clinical circumstances, and observed clinical course without anti-TB medication.

## Discussion

In this study, we showed the discordance proportion (16.9%) in molecular diagnostic methods for PTB, real-time TB/NTM PCR and Xpert MTB/RIF, and their complementary diagnostic yields using bronchial washing fluid, even though real-time TB/NTM PCR showed higher false positivity. As far as we know, this is the first study that evaluated the diagnostic yields of both assays tested with a sufficient number of bronchial washing specimens.

The sensitivity and specificity of Xpert MTB/RIF assay for diagnosis of PTB based on culture positivity were higher than that of TB/NTM PCR, and specificity was also higher in Xpert MTB/RIF assay when cases of PTB including all patients who clinically diagnosed PTB (Table 2). There is a difference between the process of Advansure TB/NTM real-time PCR and Xpert MTB/RIF assay. Xpert is an automated molecular test in which its plastic cartridge included all necessary reaction solvent for cell lysis, DNA extraction, amplification, and detection. When the specimen is inserted to the cartridge and into the GeneXpert machine, PCR is initiated automatically. However, Advansure TB/NTM real-time PCR go through course of several steps and not automatically processed. Thus, it is time consuming and may develop unintentional errors, such as inappropriately diluted specimen and cross-contamination. We showed the discordance of the two assay was not associated with any common clinical variables of PTB patients, thus,we believe that these interesting findings of this study mainly caused by not clinical characteristics of patients but by properties of the two different molecular tests itself.

Because only bronchoscopic washing specimens were collected in this study, it can reduce the heterogeneity of specimens and lead to a better diagnostic yield without any sample bias. We also performed real-time TB/NTM PCR and Xpert MTB/RIF assay simultaneously with the same specimens, and the results were free from the biases related to interpersonal variables including age, sex, host immunity, bacterial burden, and comorbidities. Even from the results analyzed without interference of host factors, we were not able to find any plausible risk factor for explaining the discordance of the two molecular methods in PTB. This finding may be important circumstantial evidence that the molecular methods can be applicable, if indicated, without bias from host conditions.

Since public screening campaigns are conducted in developed and developing countries to eradicate PTB, molecular diagnostic methods can be used more frequently in clinical fields, and this will aid in the early diagnosis of PTB. Without extensive PTB, diagnostic bronchoscopic examinations may be mandatory for collecting respiratory specimens. In this study, the sensitivity of Xpert MTB/RIF assay (85.94%) and TB/NTM PCR (92.19%) were lower than that of a recent studies. [16–18] even after analyzed including all clinically diagnosed PTB patients regardless of AFB culture positivity, 81.54% and 80%, respectively. Considering only 10 bronchoscopically obtained specimens (7.69%) showed a positive AFB smear, this suggests that majority of the study population was not able to expectorate sputum adequately or had non-extensive involvement of PTB. Therefore, under these conditions, the results of this study can be valuable for the application of molecular diagnostic methods.

As we used only a kit of AdvanSure TB/NTM real-time PCR, one of various commercial kits of real-time TB PCR, there may be an argument on the generalization of the major findings of this study. However, the sensitivity, specificity, PPV, and NPV of TB/NTM real-time PCR kit can be comparable with previous reports tested with other commercial TB PCR kits in the present study. [6,16,20–22] Moreover, the diagnostic accuracy of Xpert MTB/RIF assay in this study was accorded to the alleged diagnostic sensitivity and specificity of Xpert MTB/RIF assay on not only sputum but bronchoscopy specimens as well, which has been reported to be approximately 60–94% sensitive and 98–100% specific. [8,23–25]

Despite the high accuracy of both assays, when the assays are tested sequentially, an additional diagnostic yield was found, especially in culture-negative PTB (Fig 3). Therefore, this suggests that an additional molecular method could be helpful for patient suspected of clinical PTB. However, in this study, we should also consider that the discordance proportion was 16.9%, which is somewhat high in both PCR. The additional diagnostic gain was different in culture-confirmed PTB and culture-negative PTB. The diagnostic yields of Xpert MTB/RIF assay seemed to be more outstanding in culture-confirmed patients than in those using TB/NTM PCR, and the statistical significance was not achieved (Fig 2). Considering the finding that false positive cases were more common in real-time TB/NTM PCR and the highest discordance proportion was in patients with culture-negative and TB/NTM PCR positive results (Table 3), the culture-negative PTB group may have included non-PTB cases (i.e., false-positive cases), and it may overestimate the additional yield of real-time TB/NTM PCR. We also should consider the cost benefits of the assays, which were not elucidated in this study. Because of the high cost of Xpert MTB/RIF assay, Korean guidelines recommend that the Xpert MTB/RIF assay should be used in patients suspected of PTB and those who are at a high risk of MDR-TB, such as previously-treated patients or those with human immunodeficiency. [26] Nevertheless, in this study, the Xpert MTB/RIF assay showed higher sensitivity and additional diagnostic yield in culture-positive PTB and lower number of false-positive results. The points may be also should be considerable in clinical practice.

Despite the interesting findings, this study has some limitations. First, this retrospective study included only patients whose respiratory specimen requested for both TB/NTM PCR and Xpert MTB/RIF assay simultaneously. In majority of the cases, bronchoscopy was performed because negative results were obtained in the sputum smear tests. Only a small proportion of the study population showed positive results in the AFB smear, which indicates the possibility of selection bias that patients who could not expectorate sputum effectively or the extent of PTB might not be extensive. Secondly, this study included only a certain type of real-time PCR, and it may be necessary to test more commercial assays under local conditions. Third, we did not conduct a cost-benefit analysis in this study.

Conclusively, Xpert MTB/RIF and real-time TB/NTM PCR assay in bronchial washing specimen can provide additional diagnostic yields for PTB, although there were considerable discordant results without evidence of any risk factor related to patients. Therefore, molecular methods can be used without bias from host conditions in bronchial washing specimens.

## Supporting Information

S1 Fig Diagnostic probability of TB according to the application order of real time TB/NTM PCR or Xpert MTB/RIF assay in patients with PTB. (TIF)
