# Supplementary figures and images for: Discordance between MTB/RIF and Real-Time Tuberculosis-Specific Polymerase Chain Reaction Assay in Bronchial Washing Specimen and Its Clinical Implications
Source: PLoS One. 2016 Oct 19;11(10):e0164923. doi: 10.1371/journal.pone.0164923 (PMC5070776; doi:10.1371/journal.pone.0164923)

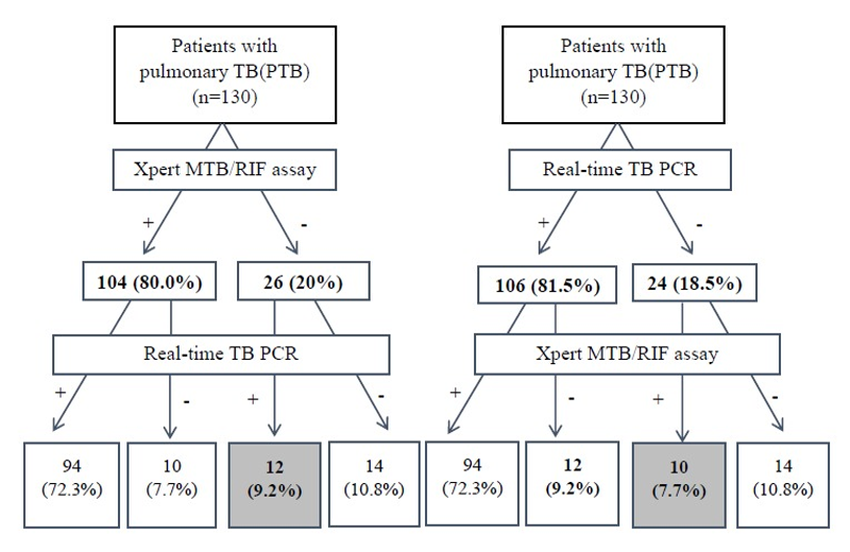

Supplement: S1 Fig — (TIF) [file pone.0164923.s001.tif]
